# A nomogram prediction model for perioperative heart failure in elderly patients with intertrochanteric femoral fracture based on lasso-logistic regression

**DOI:** 10.3389/fcvm.2026.1814710

**Published:** 2026-08-20

**Authors:** Qin Su, Yongchao Wang, Zaitian Zhang, Haibin Tang

**Affiliations:** 1 Department of Orthopedics, The First Affiliated Hospital of Hunan University of Medicine, Huaihua, Hunan, China; 2 Department of Orthopedics, Hunan University of Medicine General Hospital, Huaihua, Hunan, China

**Keywords:** elderly, heart failure, influencing factors, intertrochanteric femoral fracture, nomogram, perioperative period

## Abstract

**Objective:**

To explore a nomogram prediction model for perioperative heart failure (HF) in elderly patients with intertrochanteric femoral fracture (ITF) based on Lasso-Logistic regression.

**Methods:**

The clinical data of 345 elderly patients with ITF admitted to our hospital were retrospectively collected (as the training set). In addition, the clinical data of 251 elderly patients with ITF admitted to Hunan University of Medicine General Hospital were collected (as the validation set). Patients were separated into the HF group and the non-HF group according to whether HF occurred during the perioperative period. The clinical data of patients were collected.

**Results:**

According to Lasso-Logistic analysis, age, history of heart disease, Garden typing, preoperative electrolyte imbalance, 24 h positive balance of intake and output, and Hb were the influencing factors of HF occurrence during the perioperative period in elderly ITF patients (*P* < 0.05). The ROC curve showed that the AUC of this model was 0.886, the C-index was 0.857, the H-L test was *χ*^2^ = 7.643, *P* = 0.711, and its predicted risk of HF occurrence was highly consistent with the actual result. The DCA curve showed a relatively good positive net benefit within the probability range of 0.15∼0.86. The external validation ROC curve showed that the AUC of this model was 0.911, the C-index was 0.834, the H-L test was *χ*^2^ = 7.564, *P* = 0.705, and the predicted risk of HF was highly consistent with the actual result. The DCA curve showed a good positive net benefit within the probability range of 0.15∼0.73.

**Conclusion:**

The nomogram model constructed based on Lasso-Logistic regression in this study can assist clinicians in assessing the risk of HF in elderly ITF patients during the perioperative period, thereby implementing effective preventive measures.

## 1 Introduction

With the intensification of population aging, health issues among older adults have attracted widespread attention. Declines in limb mobility and balance in elderly individuals markedly increase the risk of osteoporosis and falls, which can lead to hip fractures. Intertrochanteric femoral fracture (ITF) is one of the common types, with an incidence that has been increasing year by year (1). ITF mainly occurs between the basal region of the femoral neck and the inferior margin of the lesser trochanter. Because the bone quality in the trochanteric region is relatively weak, fractures readily occur under external force. Once a fracture occurs, patients often require prolonged bed rest and immobilization; combined with stress and other factors, this can easily lead to heart failure (HF), with an incidence of up to 10%–27% (2, 3). Moreover, most patients have underlying diseases prior to fracture, and perioperative factors such as electrolyte disturbances may also induce HF, thereby increasing mortality and disability rates (4). Therefore, identifying factors associated with perioperative HF and implementing early prevention in clinical practice can improve patient prognosis. Lasso–Logistic regression can be used clinically to screen influencing factors: Lasso is first applied to select predictive variables and reduce overfitting among factors, followed by Logistic analysis to predict the impact of predictors on adverse events, thereby establishing a nomogram model to estimate risk values (5, 6). Studies have shown that a nomogram constructed based on HDL-C to predict preoperative deep venous thrombosis in ITF patients can help healthcare professionals assess the likelihood of thrombosis (7). The construction of related factors and predictive models for postoperative infection after ITF indicates that postoperative infection is influenced by multiple factors; the predictive model developed in these studies shows high predictive accuracy and clinical utility, aiding early identification and intervention for high-risk patients (8). Based on the above, there are currently few reports on nomogram studies of perioperative HF in elderly ITF patients. Therefore, this study aims to explore a nomogram prediction model for perioperative HF in elderly ITF patients based on Lasso–Logistic regression.

## 2 Materials and methods

### 2.1 General information

**Figure 1:**
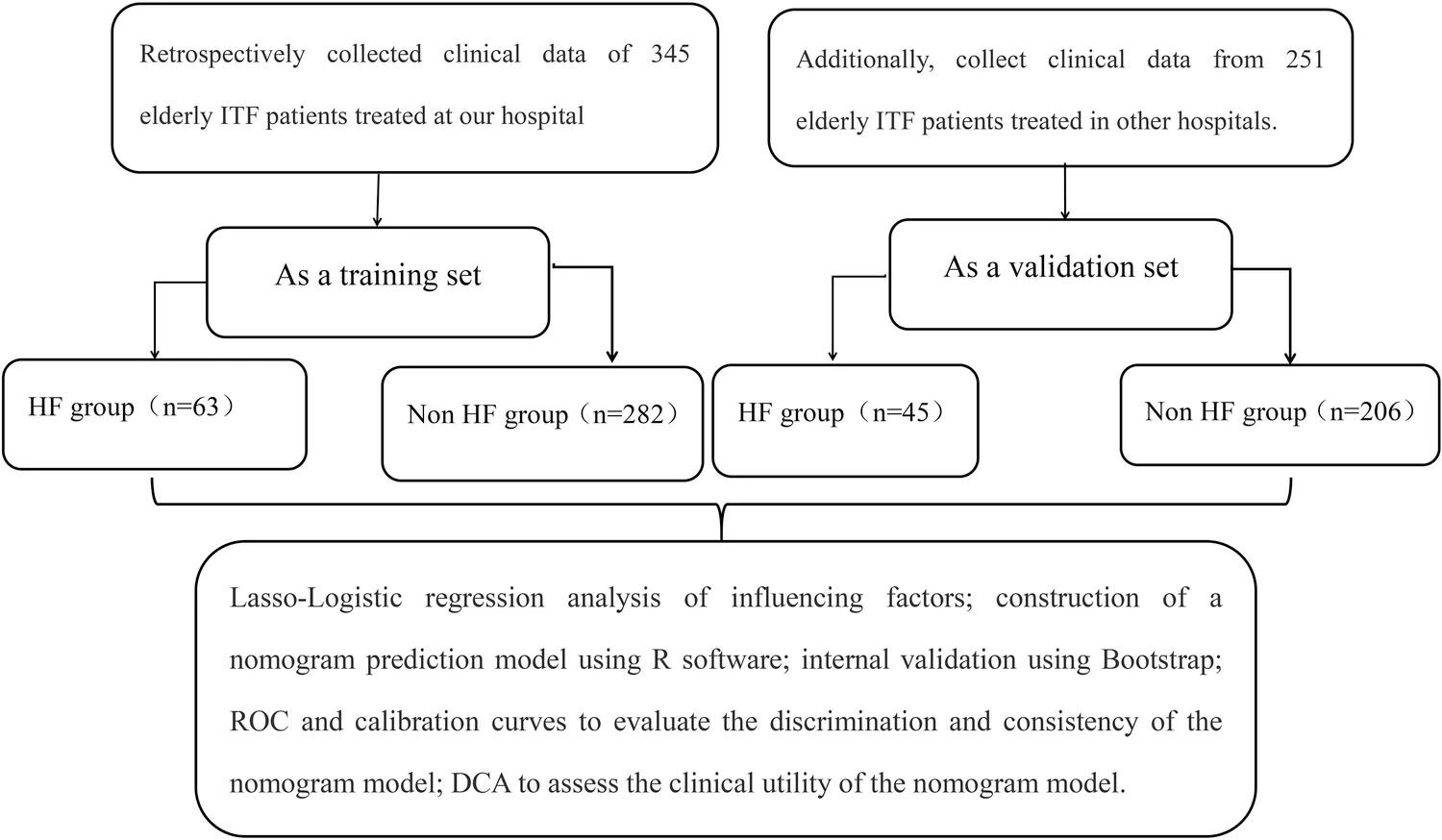
Case flow collection diagram.

Clinical data of 345 elderly ITF patients admitted to our hospital from January 2021 to January 2024 were retrospectively collected as the training set. Additionally, clinical data of 251 elderly ITF patients admitted to Hunan University of Medicine General Hospital, a general hospital in Huaihua City, Hunan Province, China, from February 2024 to September 2025 were collected as the validation set. The validation set was collected from this single hospital, and the same inclusion and exclusion criteria as those used for the training set were applied. Patients were divided into the HF group and the non-HF group according to whether HF occurred perioperatively. The case collection flowchart is shown in Figure 1. Inclusion criteria for the training and validation sets were: (1) patients clinically diagnosed with fresh closed ITF who underwent preoperative evaluation and completed reduction and internal fixation surgery; (2) age ≥75 years; (3) meeting the diagnostic criteria for HF (9). Heart failure was diagnosed when **any two or more** of the following criteria were met: the presence of signs and symptoms of heart failure, such as chest tightness, orthopnea, pulmonary rales, and lower-extremity edema; significantly elevated serum NT-proBNP levels; pulmonary congestion or pulmonary edema on chest radiography or CT; and abnormal ventricular systolic and/or diastolic function on echocardiography; (4) surgery and perioperative management performed by the same medical team; (5) complete clinical data. Exclusion criteria were: (1) pathological fractures; (2) malignant tumors; (3) injury to vital organs such as the heart or liver; (4) multiple fractures at other sites; (5) neurovascular injury; (6) severe respiratory diseases; (7) presence of non-cardiogenic factors: pneumonia was defined by focal pulmonary consolidation on chest imaging accompanied by elevated inflammatory markers; pulmonary embolism was defined by a filling defect in the pulmonary artery accompanied by elevated D-dimer levels; acute exacerbation of COPD was defined by wheezing with carbon dioxide retention and a favorable response to bronchodilator therapy; and isolated postoperative hypoxemia was defined as a transient decrease in oxygen saturation after surgery, without elevated BNP levels or abnormalities in cardiac structure or function, with rapid recovery after analgesia and oxygen therapy. This study was approved by the hospital ethics committee.

### 2.2 Clinical data

Clinical data were collected from the hospital electronic medical record system, including age, sex, preoperative ASA classification, chronic kidney disease, hypertension, diabetes mellitus, history of heart disease, cerebral infarction, preoperative NYHA classification, Garden fracture classification, preoperative electrolyte disturbance, anesthesia method, internal fixation method, infusion of human serum albumin, constipation, cognitive impairment, time from injury to surgery, operation time, perioperative blood loss, positive 24-h fluid balance, hemoglobin (Hb), left ventricular ejection fraction (LVEF), albumin (ALB), and white blood cell count (WBC). Variables with a missing data rate exceeding 5% were excluded to ensure the reliability and rigor of the results.

### 2.3 Statistical analysis

SPSS 25.0 was used for data analysis. Measurement data were analyzed using the t test and are expressed as ($\bar{x}\pm s$). Categorical data were analyzed using the *χ*^2^ test and are expressed as *n* (%). Lasso–Logistic regression was applied to analyze factors influencing perioperative HF in elderly ITF patients. R software was used to construct the nomogram prediction model for perioperative HF. Internal validation was performed using the Bootstrap method. ROC curves and calibration curves were used to evaluate the discrimination and consistency of the nomogram model. Decision curve analysis (DCA) was used to assess the clinical application value of the model. A *P* value <0.05 was considered statistically significant.

## 3 Results

### 3.1 Comparison of clinical data between HF and non-HF groups in the training set

There were significant differences between the HF and non-HF groups in age, history of heart disease, preoperative NYHA classification, Garden classification, preoperative electrolyte disturbance, positive 24-h fluid balance, and Hb (*P* < 0.05). No significant differences were found in other clinical variables (*P* > 0.05). See Table 1.

**Table 1:** Comparison of clinical data between HF and non-HF groups in the training set.

Factor	HF group (*n* = 63)	Non HF group (*n* = 282)	*t/χ* ^2^	*P*
Age (years old)	88.95 ± 5.68	82.85 ± 4.12	9.853	＜0.001
gender			0.981	0.322
male	18 (28.57)	64 (22.70)		
female	45 (71.43)	218 (77.30)		
Preoperative ASA Classification			2.715	0.099
Grade I∼II	29 (46.03)	162 (57.45)		
Grade III∼IV	34 (53.97)	120 (42.55)		
Chronic kidney disease			0.075	0.784
yes	28 (44.44)	120 (42.55)		
no	35 (55.56)	162 (57.45)		
Hypertension			0.786	0.375
yes	45 (71.43)	185 (65.60)		
no	18 (28.57)	97 (34.40)		
Diabetes			1.501	0.220
yes	28 (44.44)	102 (36.17)		
no	35 (55.56)	180 (63.83)		
History of heart disease			25.636	＜0.001
yes	43 (68.25)	95 (33.69)		
no	20 (31.75)	187 (66.31)		
Cerebral infarction			0.492	0.483
yes	15 (23.81)	56 (19.86)		
no	48 (76.19)	226 (80.14)		
Preoperative NYHA Classification			6.747	0.009
Grade I∼II	21 (33.33)	145 (51.42)		
Grade III∼IV	42 (66.67)	137 (48.58)		
Garden Typing			23.003	＜0.001
Type I∼II	17 (26.98)	170 (60.28)		
Type III∼IV	46 (73.02)	112 (39.72)		
Preoperative electrolyte imbalance			28.481	＜0.001
yes	52 (82.54)	128 (45.39)		
no	11 (17.46)	154 (54.61)		
Anesthesia method			3.592	0.058
General anesthesia	36 (57.14)	124 (43.97)		
Not general anesthesia	27 (42.86)	158 (56.03)		
Internal fixation method			0.194	0.660
Internal fixation with steel plate	21 (33.33)	86 (30.50)		
Intramedullary nailing fixation	42 (66.67)	196 (69.50)		
Infusion of human albumin			0.523	0.470
yes	38 (60.32)	156 (55.32)		
no	25 (39.68)	126 (44.68)		
Constipation			0.998	0.318
yes	19 (30.18)	68 (24.11)		
no	44 (69.84)	214 (75.89)		
Cognitive impairment			0.158	0.691
yes	16 (25.40)	65 (23.05)		
no	47 (74.60)	217 (76.95)		
Time from injury to surgery (d)	3.89 ± 0.45	3.92 ± 0.52	0.424	0.672
Surgery time (min)	180.58 ± 24.15	178.32 ± 26.45	0.623	0.534
Perioperative blood loss (mL)	1,214.68 ± 310.15	1,209.56 ± 246.25	0.142	0.887
24 h positive balance of intake and output	1,298.62 ± 104.15	946.25 ± 98.65	25.371	＜0.001
Hb (g/L)	85.69 ± 4.96	97.56 ± 5.16	16.622	＜0.001
LVEF (%)	54.69 ± 9.85	56.43 ± 10.42	1.210	0.227
ALB (g/L)	31.25 ± 5.21	32.61 ± 5.19	1.879	0.061
WBC (×10^9^/L)	11.15 ± 2.15	10.98 ± 2.46	0.507	0.613

### 3.2 Lasso–logistic regression analysis of factors influencing perioperative HF in elderly ITF patients

**Figure 2:**
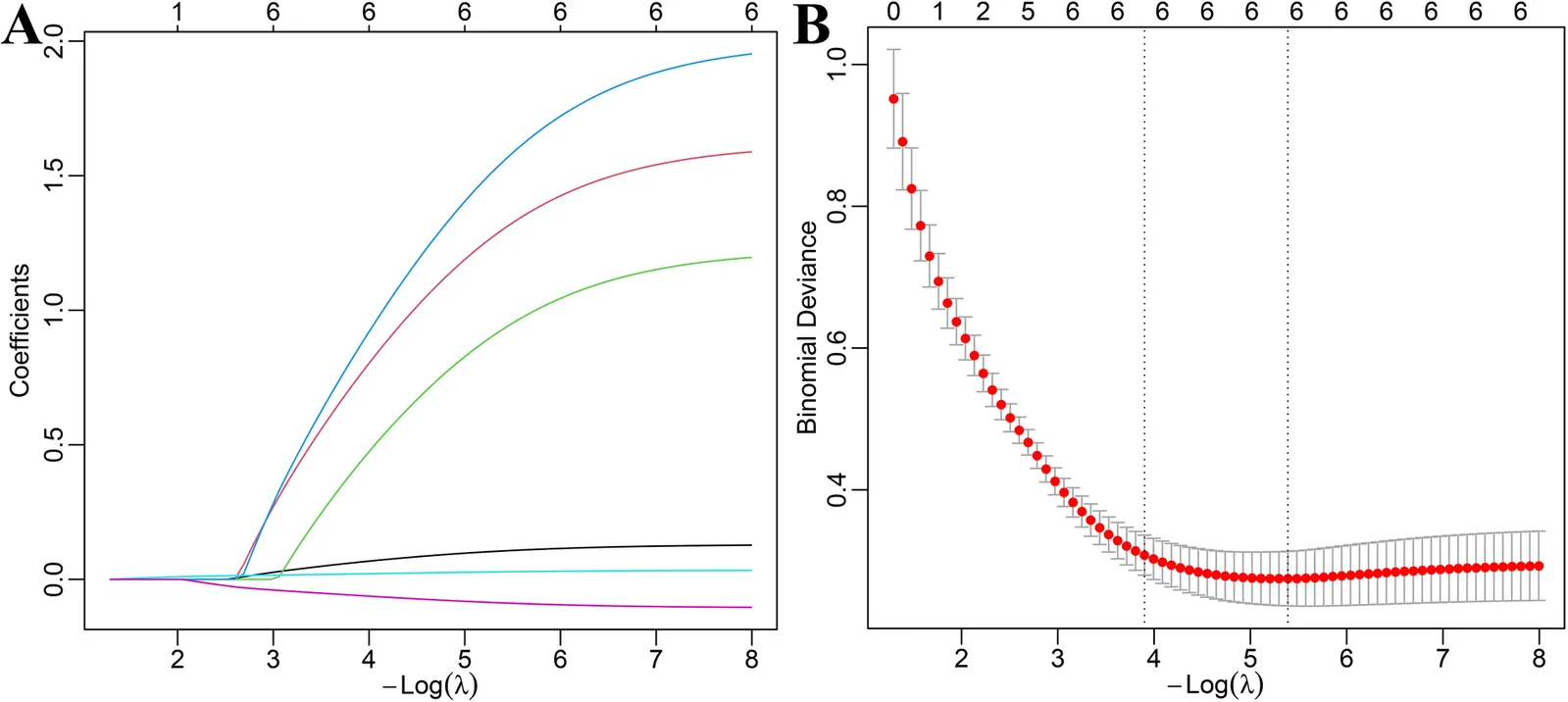
Lasso-logistic regression analysis of risk factors for perioperative heart failure in elderly patients with intertrochanteric fractures **(A)** Relationship diagram of Lasso regression coefficient; **(B)** Lasso regression 10-fold cross-validation results.

Perioperative HF in elderly ITF patients (yes=1, no = 0) was used as the dependent variable. Indicators with *P* < 0.05, including history of heart disease (yes=1, no = 0), preoperative NYHA classification (I–II = 0, III–IV = 1), Garden classification (I–II = 0, III–IV = 1), preoperative electrolyte disturbance (yes=1, no = 0), age, positive 24-h fluid balance, and Hb (entered as original values), were used as independent variables. Lasso analysis was performed using R software. When *λ*=0.02145463, six predictive factors were screened, including age, history of heart disease, Garden classification, preoperative electrolyte disturbance, positive 24-h fluid balance, and Hb (Figure 2). Multicollinearity testing of variables selected by Lasso regression showed variance inflation factors <5, indicating no interaction among factors. Logistic regression analysis (forward stepwise method) showed that age, history of heart disease, Garden classification, preoperative electrolyte disturbance, positive 24-h fluid balance, and Hb were influencing factors for perioperative HF in elderly ITF patients (*P* < 0.05). See Table 2.

**Table 2:** Lasso-Logistic regression analysis of factors affecting perioperative heart failure in elderly patients with ITF.

Variable	*Β* value	SE value	Wald$\chi^{2}$ value	*P* value	OR value	95%CI
Age	1.138	0.214	28.286	＜0.001	3.121	2.052–4.747
History of heart disease	1.516	0.648	5.463	0.019	4.554	1.278–16.231
Garden Typing	0.699	0.243	8.278	0.004	2.012	1.250–2.239
Preoperative electrolyte imbalance	0.972	0.231	30.065	＜0.001	2.643	1.681–4.157
24 h positive balance of intake and output	0.035	0.006	13.797	＜0.001	1.036	1.023–1.049
Hb	−1.064	0.218	23.831	＜0.001	0.345	0.225–0.529
constant	−37.323	8.024	21.634	＜0.001	＜0.001	-

### 3.3 Construction of the nomogram model for perioperative HF in elderly ITF patients

**Figure 3:**
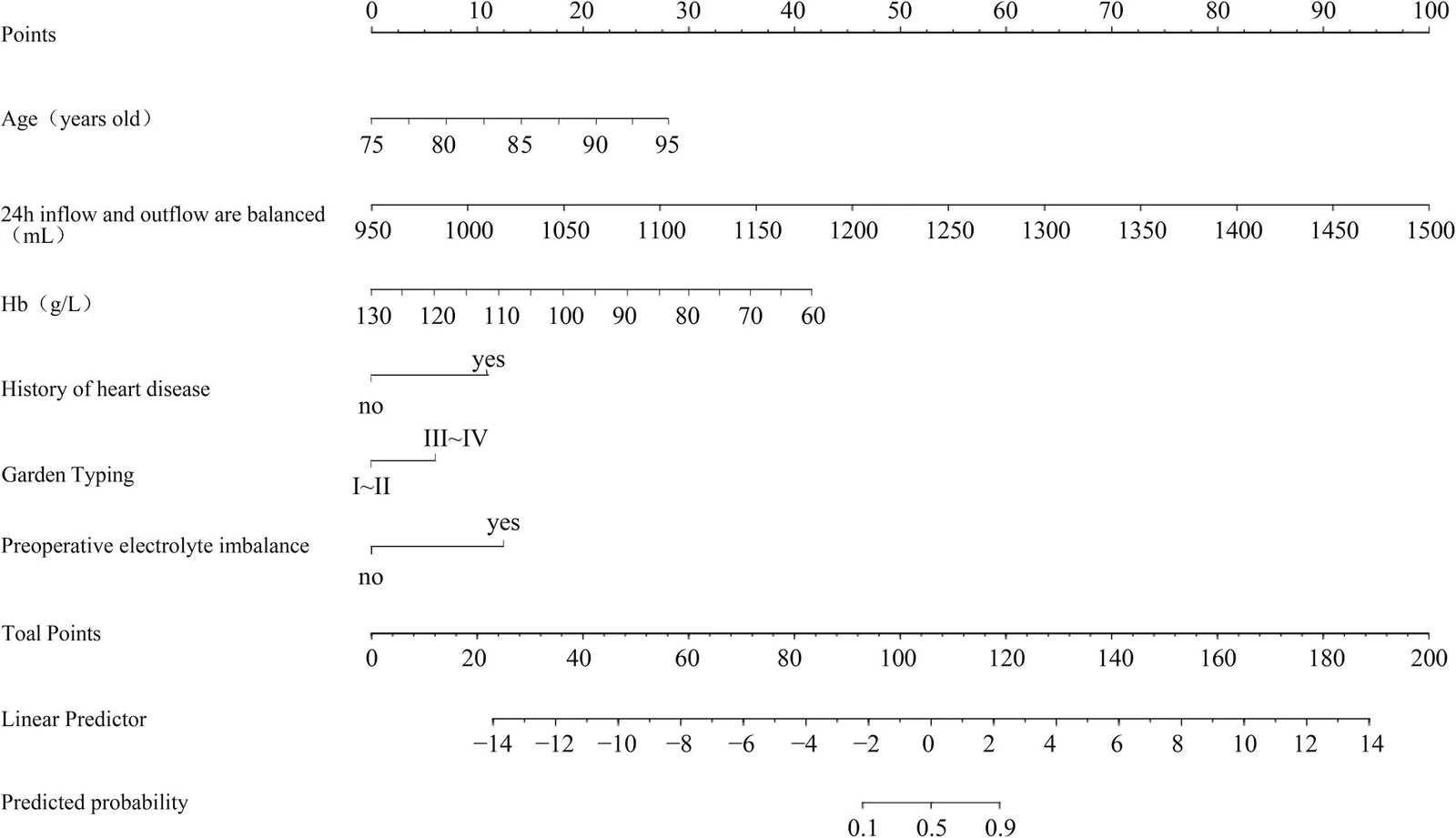
Construction of a nomogram model for perioperative heart failure in elderly ITF patients.

Based on the LASSO–Logistic regression, a nomogram model was constructed with *P* = e^x^/ (1 + e^x^), where *x* = −37.323 + 1.138×age + 1.516×history of heart disease + 0.699×Garden classification + 0.972×preoperative electrolyte disturbance + 0.035×positive 24-h fluid balance−1.064×Hb. To calculate the score, a vertical line is drawn from each variable to the Points axis, and the scores for all variables are then summed to determine the Total Points. Finally, a vertical line is drawn downward from the Total Points axis to determine the probability of HF in the patient (10, 11). For example, a total score of 116 corresponds to a 72% probability of HF. See Figure 3.

### 3.4 Internal validation of the nomogram model for perioperative HF in elderly ITF patients

**Figure 4:**
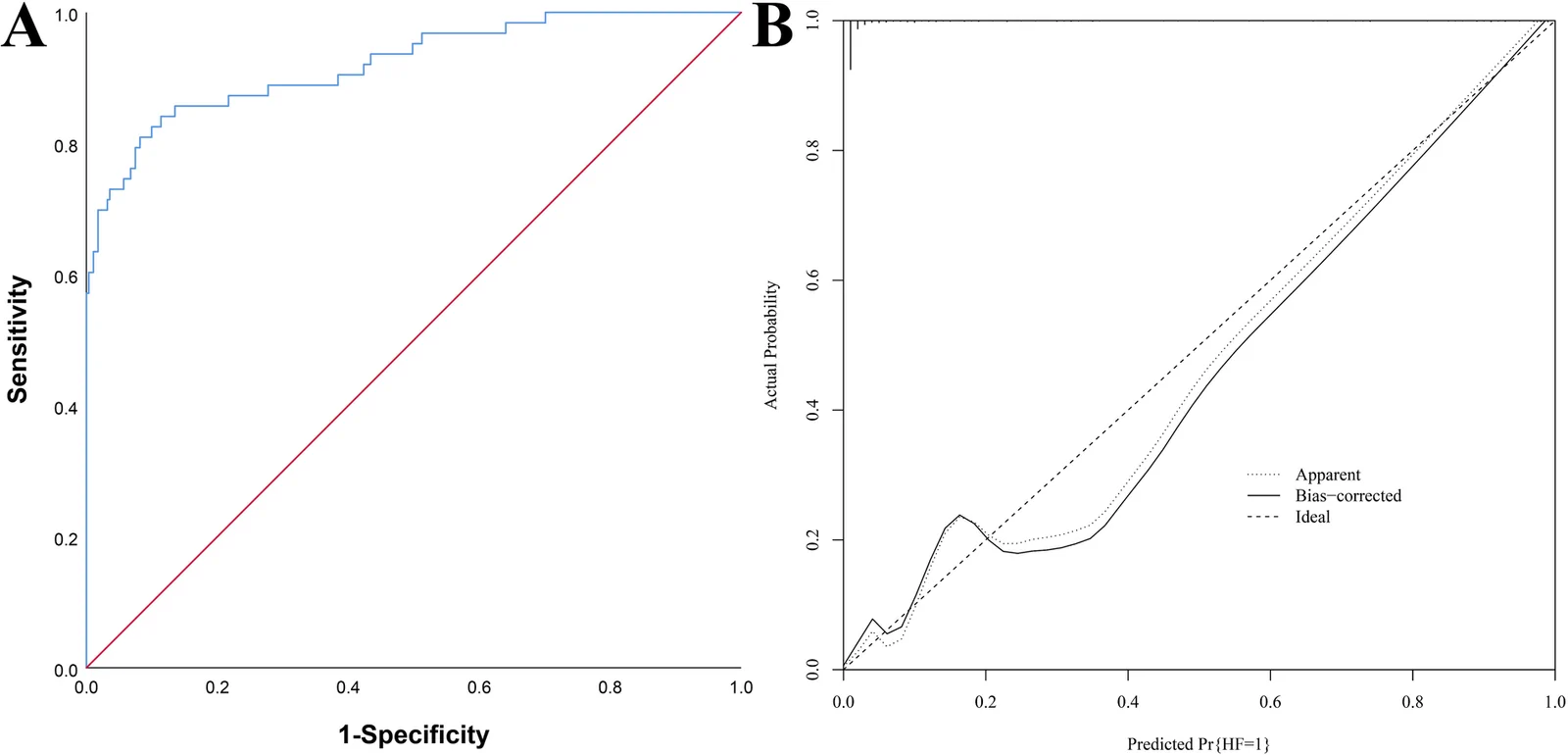
Internal validation of the nomogram predicting perioperative heart failure in elderly patients with intertrochanteric fractures **(A)** ROC curves; **(B)** Calibration curves.

The ROC curve showed that the AUC for predicting perioperative HF was 0.886 (95% CI: 0.816–0.946), with a sensitivity of 89.24% and a specificity of 80.13%. Internal validation using the Bootstrap method (1,000 repetitions) showed a C-index of 0.857. The Hosmer–Lemeshow test yielded *χ*^2^ = 7.643, *P* = 0.711, indicating high agreement between predicted HF risk and actual outcomes. See Figure 4.

### 3.5 Curve of the internally validated nomogram model

**Figure 5:**
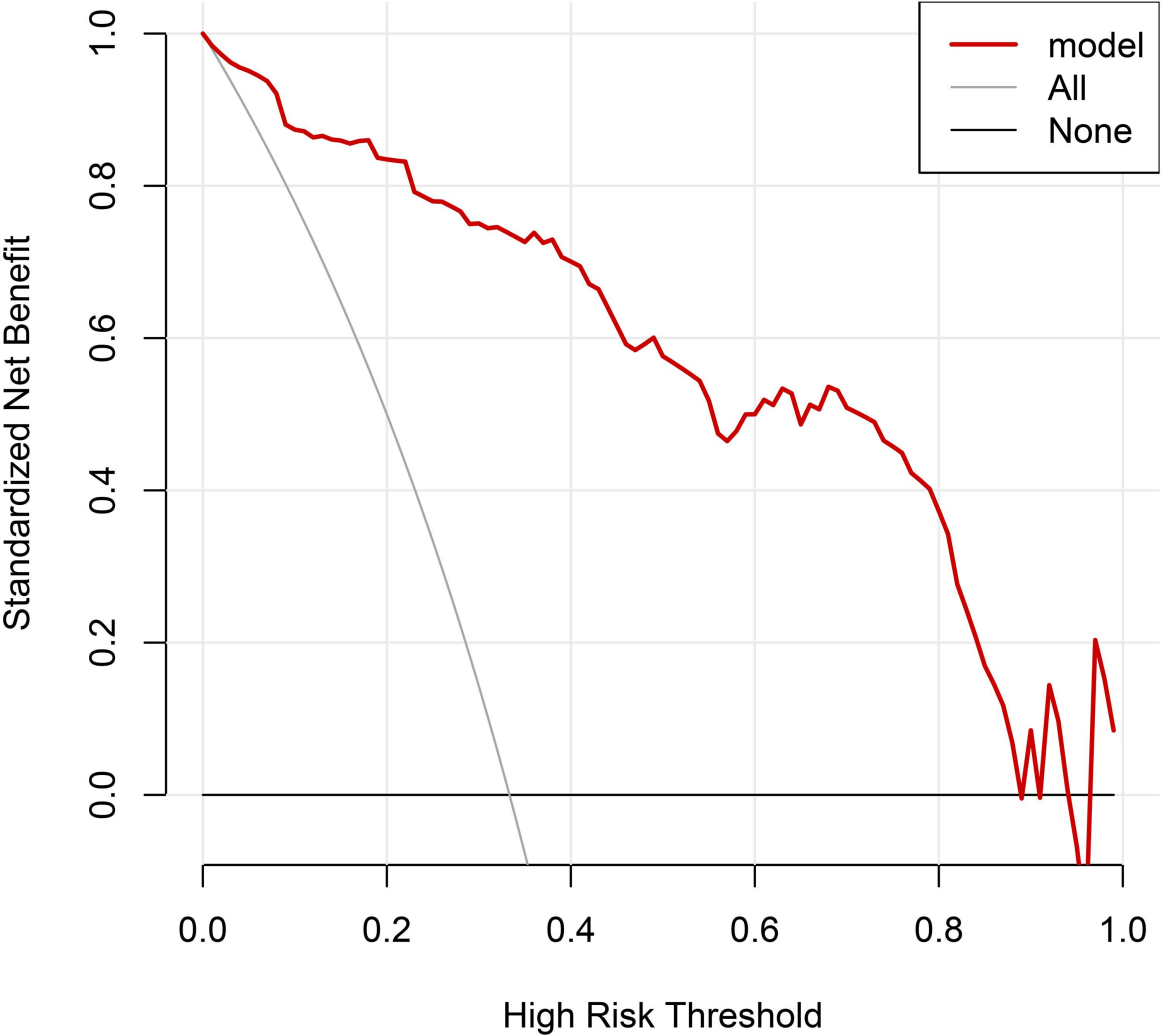
Internal validation of DCA curve for nomogram model.

The DCA curve showed good positive net benefit within a probability range of 0.15–0.86, indicating good clinical application value of the model. See Figure 5.

### 3.6 Comparison of clinical data between HF and non-HF groups in the validation set

There were significant differences between the HF and non-HF groups in age, history of heart disease, preoperative NYHA classification, Garden classification, preoperative electrolyte disturbance, positive 24-h fluid balance, and Hb (*P* < 0.05). No significant differences were observed for other variables (*P* > 0.05). See Table 3.

**Table 3:** Comparison of clinical data between HF and non-HF groups in the validation set.

Factor	HF group (*n* = 45)	Non HF group (*n* = 206)	*t/χ* ^2^	*P*
Age (years old)	89.59 ± 5.45	83.16 ± 5.33	7.302	＜0.001
gender			0.489	0.484
male	12 (26.67)	45 (21.84)		
female	33 (73.33)	161 (78.16)		
Preoperative ASA Classification			1.198	0.274
Grade I∼II	21 (46.67)	78 (37.86)		
Grade III∼IV	24 (53.33)	128 (62.14)		
Chronic kidney disease			0.110	0.740
yes	20 (44.44)	86 (41.75)		
no	25 (55.56)	120 (58.25)		
Hypertension			0.773	0.379
yes	34 (75.56)	142 (68.93)		
no	11 (24.44)	64 (31.07)		
Diabetes			0.015	0.904
yes	19 (42.22)	89 (43.20)		
no	26 (57.78)	117 (56.80)		
History of heart disease			19.980	＜0.001
yes	30 (66.67)	64 (31.07)		
no	15 (33.33)	142 (68.93)		
Cerebral infarction			0.687	0.407
yes	10 (22.22)	35 (16.99)		
no	35 (77.78)	171 (83.01)		
Preoperative NYHA Classification			5.842	0.016
Grade I∼II	14 (31.11)	105 (50.97)		
Grade III∼IV	31 (68.89)	101 (49.03)		
Garden Typing			16.495	＜0.001
Type I∼II	14 (31.11)	132 (64.08)		
Type III∼IV	31 (68.89)	74 (35.92)		
Preoperative electrolyte imbalance			27.697	＜0.001
yes	35 (77.78)	72 (34.95)		
no	10 (22.22)	134 (65.05)		
Anesthesia method			0.539	0.463
General anesthesia	25 (55.56)	102 (49.51)		
Not general anesthesia	20 (44.44)	104 (50.49)		
Internal fixation method			0.011	0.917
Internal fixation with steel plate	15 (33.33)	67 (32.52)		
Intramedullary nailing fixation	30 (66.67)	139 (67.48)		
Infusion of human albumin			0.024	0.876
yes	27 (60.32)	121 (58.74)		
no	18 (40.00)	85 (4,126)		
Constipation			0.158	0.691
yes	14 (31.11)	58 (28.16)		
no	31 (68.89)	148 (71.84)		
Cognitive impairment			0.072	0.789
yes	12 (26.67)	51 (24.76)		
no	33 (73.33)	155 (75.24)		
Time from injury to surgery (d)	3.91 ± 0.48	3.78 ± 0.46	1.704	0.090
Surgery time (min)	180.46 ± 23.16	179.51 ± 24.25	0.240	0.811
Perioperative blood loss (mL)	1,209.74 ± 306.55	1,206.54 ± 286.52	0.062	0.947
24 h positive balance of intake and output	1,189.52 ± 103.45	951.62 ± 99.43	14.436	＜0.001
Hb (g/L)	84.23 ± 4.85	98.26 ± 5.01	17.114	＜0.001
LVEF (%)	55.12 ± 9.42	56.38 ± 10.42	0.747	0.456
ALB (g/L)	32.32 ± 5.14	32.24 ± 5.21	0.094	0.926
WBC (×10^9^/L	11.13 ± 2.04	10.86 ± 2.01	0.814	0.416

### 3.7 External validation of the nomogram model for perioperative HF in elderly ITF patients

**Figure 6:**
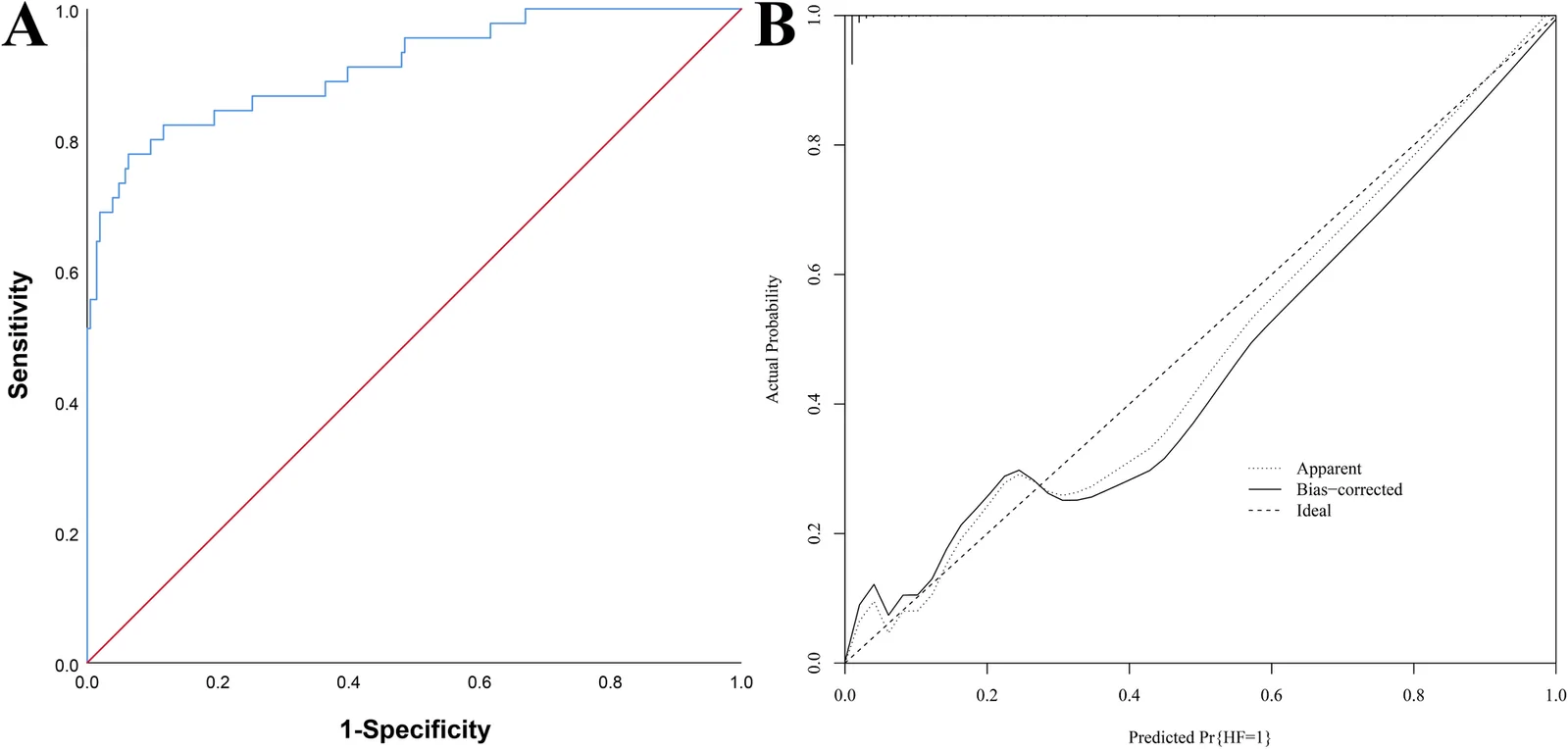
External validation of the nomogram predicting perioperative heart failure in elderly patients with intertrochanteric fractures. **(A)** ROC curves; **(B)** Calibration curves.

The ROC curve showed that the AUC for predicting perioperative HF was 0.911 (95% CI: 0.859–0.964), with a sensitivity of 82.28% and a specificity of 81.34%. Bootstrap internal validation (1,000 repetitions) showed a C-index of 0.834. The Hosmer–Lemeshow test yielded *χ*^2^ = 7.564, *P* = 0.705, indicating high agreement between predicted HF risk and actual outcomes. See Figure 6.

### 3.8 Curve of the externally validated nomogram model

**Figure 7:**
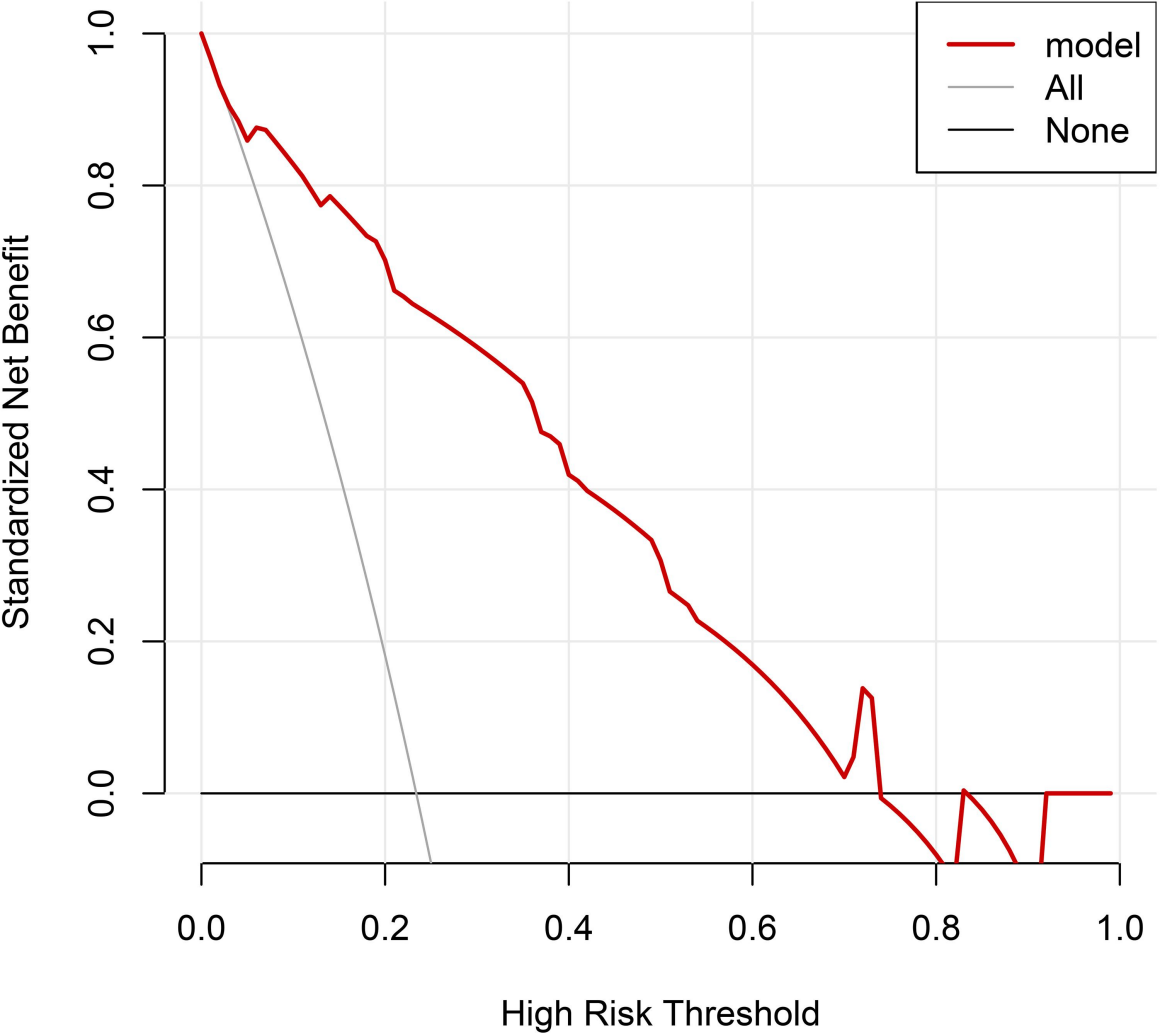
External validation of the nomogram model DCA curve.

The DCA curve showed good positive net benefit within a probability range of 0.15–0.73, indicating good clinical application value of the model. See Figure 7.

## 4 Discussion

Statistics indicate that approximately 1.6 million patients worldwide experience hip fractures each year, with a year-by-year increase among elderly individuals. As one type of hip fracture, ITF often occurs in older patients with comorbid medical conditions, increasing treatment difficulty. Conservative treatment can be effective for some ITF patients, but prolonged bed rest may lead to cardiopulmonary complications. It is widely accepted that early surgery and early mobilization can reduce related complications and mortality (12, 13). Surgical treatment itself is traumatic and may exacerbate pre-existing conditions such as hyperglycemia and HF during the perioperative period; moreover, patients with fractures complicated by HF have a significantly increased risk of death (14). In this study, the incidence of perioperative HF in elderly ITF patients was 18.26% (63/345), similar to the previously reported 18.37% (138/751) (15), indicating a relatively high incidence. Therefore, identifying factors associated with perioperative HF and promptly screening high-risk populations for intervention is clinically important.

Lasso–Logistic analysis in this study showed that age, history of heart disease, Garden classification, preoperative electrolyte disturbance, positive 24-h fluid balance, and Hb were influencing factors for perioperative HF in elderly ITF patients. The underlying reasons are as follows. (1) In very elderly patients, organ function declines; combined with the stress of trauma and anesthesia, systemic deterioration may occur, triggering latent diseases. Elderly individuals also exhibit reduced cardiac compliance and reserve capacity, leading to diminished compensatory ability. Perioperative changes in internal environment and cardiac preload and afterload can damage myocardial cells, resulting in decompensated cardiac function and HF, especially in patients aged >80 years, who exhibit marked degenerative changes in cardiomyocytes, substantially reduced cardiac systolic and diastolic reserve, decreased vascular elasticity, and extremely poor tolerance to perioperative stress; even minor fluctuations in volume load may increase the risk of HF (16, 17). Thus, special attention should be paid to very elderly patients. (2) In patients with a history of heart disease(chronic heart failure, previous myocardial infarction/coronary heart disease, persistent atrial fibrillation, hypertensive heart disease, and valvular heart disease were considered high-risk types), long-term impairment of cardiac function combined with trauma and surgical stress reduces cardiac pumping ability, aggravates cardiac dysfunction, and leads to decompensation; such patients are more prone to perioperative HF (15, 18). Therefore, preoperative cardiac risk stratification may be performed in patients with the aforementioned structural heart diseases, with enhanced perioperative cardiac function monitoring and fluid management. (3) Garden classification is commonly used clinically for femoral fractures; higher grades indicate more severe impairment of femoral head blood supply. In this study, patients with type III or above were more likely to develop perioperative HF, mainly because unstable fractures cause greater soft tissue damage and more severe impairment of femoral head blood flow. In elderly patients with poor nutrition, increased vascular fragility, and low pain tolerance, organ function may deteriorate, leading to hemodynamic instability and abnormal cardiac pump function, thereby inducing HF (19). (4) Preoperative electrolyte disturbances can markedly increase levels of prostaglandins and catecholamines in elderly patients, causing internal environment imbalance and multiple adverse reactions, thereby triggering HF (4, 20). (5) After fracture, patients may experience blood and fluid loss due to surgery, necessitating fluid replacement to maintain organ perfusion. However, excessive or rapid perioperative fluid infusion can sharply increase circulating blood volume and cardiac preload in a short time, exceeding the cardiac compliance of elderly patients and ultimately inducing HF (21). (6) Reduced preoperative Hb levels decrease the oxygen-carrying capacity of red blood cells, leading to tissue hypoxia, which promotes NO release and activates the sympathetic nervous system, resulting in sodium and water retention. Red blood cells contain antioxidants; anemia increases oxidative stress and free radical production, causing myocardial injury and leading to HF. In this study, we found that when the Hb level was below the cutoff value of 80.12 g/L, reduced myocardial oxygen supply combined with a hyperdynamic circulatory state significantly increased the risk of perioperative HF (22, 23).

The nomogram model developed in this study showed AUC values of 0.857 and 0.911 and C-indices of 0.857 and 0.834 in the training and validation sets, respectively, indicating good consistency and high agreement between predicted HF risk and actual outcomes. The DCA curves showed that the model had high clinical value within threshold probability ranges of 0.15–0.86 and 0.15–0.73, respectively, and can assist clinicians in early prevention and improvement of patient prognosis.

In summary, the nomogram model based on Lasso–Logistic regression constructed in this study can assist clinicians in assessing the risk of perioperative HF in elderly ITF patients and implementing effective preventive measures. This study has limitations, including a relatively small sample size and its retrospective design. Future studies will expand the sample size and conduct prospective validation.

## Data Availability

The original contributions presented in the study are included in the article/Supplementary Material, further inquiries can be directed to the corresponding author.

## References

[1] Fischer H Maleitzke T Eder C Ahmad S Stöckle U Braun KF. Management of proximal femur fractures in the elderly: current concepts and treatment options. Eur J Med Res. (2021) 26(1):86. 10.1186/s40001-021-00556-0 34348796 PMC8335457

[2] Bedrettin A Sahin F Yucel M O. Treatment of intertrochanteric femur fracture with closed external fixation in high-risk geriatric patients: can it be the most reliable method that reduces mortality to minimum compared to proximal femoral nail and hemiarthroplasty? Medicine (Baltimore). (2022) 101(1):e28369. 10.1097/MD.0000000000028369 35029883 PMC8735793

[3] Jürgens L Sarabhai T Kostev K. In-Hospital mortality among elderly patients hospitalized for femur fracture with and without diabetes Mellitus: a multicenter case-control study. J Clin Med. (2024) 13(21):6484. 10.3390/jcm13216484 39518624 PMC11546991

[4] Vitiello R Pesare E Capece G Di Gialleonardo E De Matthaeis A Franceschi F. Surgical timing and clinical factor predicting in-hospital mortality in older adults with hip fractures: a neuronal network analysis. J Orthop Traumatol. (2025) 26(1):30. 10.1186/s10195-025-00846-x 40369316 PMC12078743

[5] Gou D Min C Peng X Wu H Zhang L Chen Y. Associating factors of cognitive frailty among older people with chronic heart failure: based on LASSO-logistic regression. J Adv Nurs. (2024) 81(3):1399–411. 10.1111/jan.16352 39078209

[6] Wang S Bai G-L Wang Z-H. Comment on ‘associating factors of cognitive frailty among older people with chronic heart failure: based on LASSO-logistic regression’. J Adv Nurs. (2025) 81(12):8999–9000. 10.1111/jan.16825 39953805

[7] Li W Ling H Lu R Huang Z Su W. Nomogram based on high-density lipoprotein cholesterol for the occurrence of preoperative deep vein thrombosis in patients with intertrochanteric femur fracture: a retrospective study. J Orthop Surg Res. (2024) 19(1):22. 10.1186/s13018-023-04497-8 38167173 PMC10763374

[8] Xi Y-L Li B Pu L-Q Ma T Luo X-P. Factors associated with the occurrence of postoperative infection of intertrochanteric fracture of the femur and its prediction model creation and validation. Medicine (Baltimore). (2025) 104(29):e43397. 10.1097/MD.0000000000043397 40696658 PMC12282799

[9] Dunlay SM Givertz MM Aguilar D Allen LA Chan M Desai AS. Type 2 diabetes Mellitus and heart failure: a scientific statement from the American Heart Association and the heart failure society of America: this statement does not represent an update of the 2017 ACC/AHA/HFSA heart failure guideline update. Circulation. (2019) 140(7):e294–324. 10.1161/CIR.0000000000000691 31167558

[10] Wu Z Zhan S Wang D Tang C. A novel nomogram for predicting the risk of acute heart failure in intensive care unit patients with COPD. Chronic Obstr Pulm Dis. (2025) 12(2):117–26. 10.15326/jcopdf.2024.0562 39912871 PMC12147825

[11] Huang X Zhang W. Preoperative MRI-based tumor volume or tumor thickness for tongue cancer: which is more important? J Craniomaxillofac Surg. (2026) 54(9):104624. 10.1016/j.jcms.2026.104624 42258892

[12] Polat G Bayram S Gökçeoğlu YS Albayrak O Kahraman A Durmaz H. The effect of bone morphology on fracture type and treatment result in patients with intertrochanteric femur fracture aged over 65 year. Ulus Travma Acil Cerrahi Derg. (2022) 28(12):1731–8. 10.14744/tjtes.2022.57400 36453791 PMC10198310

[13] Çukurlu M Karagoz B Keceli O. The effect of pre-fracture proximal femur geometry on hip fracture type in elderly patients. Medicine (Baltimore). (2023) 102(19):e33622. 10.1097/MD.0000000000033622 37171316 PMC10174388

[14] Marco-Martínez J Bernal-Sobrino JL Fernández-Pérez C Elola-Somoza FJ Azaña-Gómez J García-Klepizg JL. Impact of heart failure on in-hospital outcomes after surgical femoral neck fracture treatment. J Clin Med. (2021) 10(5):969. 10.3390/jcm10050969 33801169 PMC7957564

[15] Tian M Li W Wang Y Tian Y Zhang K Li X. Risk factors for perioperative acute heart failure in older hip fracture patients and establishment of a nomogram predictive model. J Orthop Surg Res. (2023) 18(1):347. 10.1186/s13018-023-03825-2 37165391 PMC10170845

[16] Tamamura Y Matsuura M Shiba S Nishikimi T. Effect of heart failure and malnutrition, alone and in combination, on rehabilitation effectiveness in patients with hip fracture. Clin Nutr ESPEN. (2021) 44:356–66. 10.1016/j.clnesp.2021.05.014 34330490

[17] Fu M Zhang Y Guo J Zhao Y Hou Z Wang Z. Application of integrated management bundle incorporating with multidisciplinary measures improved in-hospital outcomes and early survival in geriatric hip fracture patients with perioperative heart failure: a retrospective cohort study. Aging Clin Exp Res. (2022) 34(5):1149–58. 10.1007/s40520-021-02038-z 35067910 PMC9135836

[18] Yu Q Fu M Hou Z Wang Z. Developing a prediction model for preoperative acute heart failure in elderly hip fracture patients: a retrospective analysis. BMC Musculoskelet Disord. (2024) 25(1):736. 10.1186/s12891-024-07843-x 39277727 PMC11401261

[19] Buckman SA Forrester JD Bessoff KE Parli SE Evans HL Huston JM. Surgical infection society guidelines: 2022 updated guidelines for antibiotic use in open extremity fractures. Surg Infect (Larchmt). (2022) 23(9):817–28. 10.1089/sur.2022.206 36350736

[20] Peng Z Wu J Wang Z Xie H Wang J Zhang P. Incidence and related risk factors for postoperative delirium following revision total knee arthroplasty: a retrospective nationwide inpatient sample database study. BMC Musculoskelet Disord. (2024) 25(1):633. 10.1186/s12891-024-07757-8 39118027 PMC11313129

[21] Yu Q Fu M Wang Z Hou Z. Predictive characteristics and model development for acute heart failure preceding hip fracture surgery in elderly hypertensive patients: a retrospective machine learning approach. BMC Geriatr. (2024) 24(1):296. 10.1186/s12877-024-04892-8 38549043 PMC10976760

[22] Riaz MH Riaz J Mahmood A Tariq M Sahar N Ali RS. Risk factors of postoperative acute heart failure in elderly patients after hip fracture surgery. Cureus. (2024) 16(4):e58967. 10.7759/cureus.58967 38800267 PMC11127705

[23] Zhao W Fu M Wang Z Hou Z. Risk factors and prognosis of perioperative acute heart failure in elderly patients with hip fracture: case-control studies and cohort study. BMC Musculoskelet Disord. (2024) 25(1):143. 10.1186/s12891-024-07255-x 38355490 PMC10868018

